# Prevalence and duration of SARS-CoV-2 fecal shedding in breastfeeding dyads following maternal COVID-19 diagnosis

**DOI:** 10.3389/fimmu.2024.1329092

**Published:** 2024-03-21

**Authors:** Ryan M. Pace, Elana A. King-Nakaoka, Andrew G. Morse, Kelsey J. Pascoe, Anna Winquist, Beatrice Caffé, Alexandra D. Navarrete, Kimberly A. Lackey, Christina D.W. Pace, Bethaney D. Fehrenkamp, Caroline B. Smith, Melanie A. Martin, Celestina Barbosa-Leiker, Sylvia H. Ley, Mark A. McGuire, Courtney L. Meehan, Janet E. Williams, Michelle K. McGuire

**Affiliations:** ^1^ Margaret Ritchie School of Family and Consumer Sciences, University of Idaho, Moscow, ID, United States; ^2^ College of Nursing, University of South Florida, Tampa, FL, United States; ^3^ University of Washington School of Medicine, Seattle, WA, United States; ^4^ WWAMI Medical Education, University of Idaho, Moscow, ID, United States; ^5^ College of Nursing, Washington State University, Spokane, WA, United States; ^6^ College of Nursing, University of Colorado Anschutz Medical Campus, Aurora, CO, United States; ^7^ Department of Anthropology, Washington State University, Pullman, WA, United States; ^8^ Department of Medicine, Oregon Health and Sciences University, Portland, OR, United States; ^9^ Department of Anthropology, University of Washington, Seattle, WA, United States; ^10^ Center for Studies in Demography and Ecology, University of Washington, Seattle, WA, United States; ^11^ School of Public Health and Tropical Medicine, Tulane University, New Orleans, LA, United States; ^12^ Department of Animal, Veterinary and Food Sciences, University of Idaho, Moscow, ID, United States

**Keywords:** breastfeeding, COVID-19, feces, lactation, maternal-infant health, SARS-CoV-2, symptoms, viral shedding

## Abstract

**Background:**

There is a paucity of data on the presence of severe acute respiratory syndrome coronavirus 2 (SARS-CoV-2) in feces of lactating women with coronavirus disease 2019 (COVID-19) and their breastfed infants as well as associations between fecal shedding and symptomatology.

**Objective:**

We examined whether and to what extent SARS-CoV-2 is detectable in the feces of lactating women and their breastfed infants following maternal COVID-19 diagnosis.

**Methods:**

This was a longitudinal study carried out from April 2020 to December 2021 involving 57 breastfeeding maternal-infant dyads: 33 dyads were enrolled within 7 d of maternal COVID-19 diagnosis, and 24 healthy dyads served as controls. Maternal/infant fecal samples were collected by participants, and surveys were administered via telephone over an 8-wk period. Feces were analyzed for SARS-CoV-2 RNA.

**Results:**

Signs/symptoms related to ears, eyes, nose, and throat (EENT); general fatigue/malaise; and cardiopulmonary signs/symptoms were commonly reported among mothers with COVID-19. In infants of mothers with COVID-19, EENT, immunologic, and cardiopulmonary signs/symptoms were most common, but prevalence did not differ from that of infants of control mothers. SARS-CoV-2 RNA was detected in feces of 7 (25%) women with COVID-19 and 10 (30%) of their infants. Duration of fecal shedding ranged from 1-4 wk for both mothers and infants. SARS-CoV-2 RNA was sparsely detected in feces of healthy dyads, with only one mother’s and two infants’ fecal samples testing positive. There was no relationship between frequencies of maternal and infant SARS-CoV-2 fecal shedding (*P*=0.36), although presence of maternal or infant fever was related to increased likelihood (7-9 times greater, *P*≤0.04) of fecal shedding in infants of mothers with COVID-19.

## 1 Introduction

During the initial phase of the coronavirus disease 2019 (COVID-19) pandemic the risk of maternal-to-child transmission of severe acute respiratory syndrome coronavirus 2 (SARS-CoV-2) was cited as one of a battery of reasons why mother-infant separation was initially recommended following maternal COVID-19 diagnosis (1). In addition to respiratory transmission, several other modes were initially of concern including via breastfeeding and fecal-oral transmission (2–6). A large body of research quickly accumulated that did not support the proposition that human milk is a common vehicle for maternal-to-infant SARS-CoV-2 transmission and is instead a source of passive immunity for infants (7–16). However, less attention has been paid to the fecal-oral route of transmission in breastfeeding dyads.

Nearly 25% of COVID-19 patients manifest gastrointestinal (GI) signs/symptoms (e.g., diarrhea, nausea, vomiting), and the presence of SARS-CoV-2 in feces is a relatively common occurrence in individuals with COVID-19, with some studies observing viral shedding in as many as 40.5% of COVID-19 patients (6, 17–21). In addition, SARS-CoV-2 RNA has been detected in feces of pre-symptomatic and asymptomatic individuals (18). Although SARS-CoV-2 RNA concentrations in feces (an estimated 10^2^-10^7^ genome copies/mL) are several orders of magnitude lower than those of nasopharyngeal (NP) fluids (10^5^-10^11^ genome copies/mL), there is evidence of viable, replication-competent, SARS-CoV-2 in fecal samples (20–24). Consequently, risk of SARS-CoV-2 transmission via the fecal-oral route is possible. It should be noted that while most of these data were obtained from adult patient populations, several infant case studies have been reported (25–36). The risk for maternal-to-child viral transmission in breastfeeding dyads may be higher than in other settings and situations as repeated close-contact interactions occur frequently between mothers and infants (37, 38). For example, Chen et al. (2020) reported on a family cluster with an asymptomatic female child (11 mo of age) who persistently tested negative via NP swab while also persistently shedding SARS-CoV-2 RNA in her feces for ≥100 d from the date of illness onset (26). In another study of three pediatric cases (1.5 to 6 yr of age), SARS-CoV-2 RNA was detectable in feces for 8 to 20 d after the children’s respiratory swabs first became negative (27). Although infants can be at risk for severe COVID-19, this remains an infrequent outcome (39, 40). Of note, whether any of the infants or older children were breastfed at the time of sampling was not indicated in any of these reports.

We previously reported on the lack of detection of SARS-CoV-2 in milk and the inconsistent detection in swabs of the nipple/areola as well as milk-borne and circulating anti-SARS-CoV-2 receptor binding domain (RBD) antibodies of lactating women with COVID-19 (10, 12, 41). In the study reported herein, we assessed the presence of SARS-CoV-2 RNA in feces collected from these women and their infants following the onset of maternal COVID-19 and in healthy controls for 8 wk. We also characterized associations between SARS-CoV-2 RNA presence in maternal and infant feces and signs and symptoms of COVID-19 in the maternal-infant dyads.

## 2 Materials and methods

### 2.1 Experimental design and clinical data/sample collection

This multicenter prospective study was carried out from April 2020 to March 2021 using a repeated-measures, longitudinal design. Maternal-infant dyads were recruited locally through participating institutions (University of Idaho, Tulane University, University of Washington, and Washington State University) and through national social media and other forms of advertising. To participate, women had to be ≥18 yr of age, lactating, and have an infant younger than 24 mo. Two groups of maternal-infant dyads were recruited (1): those in which the mother was clinically tested/diagnosed with COVID-19 in the last seven days (“COVID-19 group,” *n*=33), and (2) those with no known prior COVID-19 infections or exposures and who reported no current COVID-19 symptoms (“healthy group,” *n*=24). One dyad from the COVID-19 group included two lactating parents who both provided milk to the infant. Due to the nature of conducting this research during a pandemic and because test results were often not available soon after testing, some participants were recruited after being tested but prior to receiving results for their COVID-19 tests. Participants receiving a negative COVID-19 result after enrollment were ineligible to participate in the study. Several participating mothers reported receiving a COVID-19 vaccination, including one participant (1 mo after enrollment) from the COVID-19 group and eight participants (*n*=3, initial vaccine administered on average 9 d prior to enrollment; *n*=5, initial vaccine administered on average 20 d after enrollment) from the healthy group.

Fecal samples were collected from each mother and child in the COVID-19 group and telephone surveys administered on three separate days during the first week following enrollment (1, 2-6, and 7 d) and again around 2, 3, 4, and 8 wk following enrollment. For the healthy group, fecal samples were collected from each mother and infant and telephone surveys administered on two separate days during the first week after enrollment and again around 3 and 8 wk after enrollment. Women self-collected fecal samples using provided collection kits (Fe-Col Faeces Collection Device, cat. FC2010, Alpha Laboratories; Faeces Collection Tube, cat. 80.734.00, Sarstedt), which were assembled aseptically by study personnel wearing masks, gowns, and gloves. Infant fecal samples were collected by participants using provided fecal collection containers and spatulas (Faeces Collection Tube, cat. 80.734.00, Sarstedt); 2-4 scoops of feces were obtained from either the infant’s diaper or skin. Participants were instructed by study personnel how to use aseptic technique to obtain samples, including the use of gloves and masks. Samples were then immediately frozen in the participant’s freezer. Samples were either shipped overnight from the subject’s home to the University of Idaho (UI) in a cooler containing frozen cold packs or picked up by local study personnel and transported (or overnight shipped on dry ice) back to UI and stored at -80°C. A total of 544 fecal samples (*n*=255 maternal, *n*=289 infant) were available for analyses (**Supplementary Figure S1**, **Supplementary Table S1**).

Telephone surveys were administered by trained study personnel using a set protocol and included questions about COVID-19 clinical testing results for all household members as well as maternal and infant signs/symptoms of any illnesses or ailments (e.g., congestion, cough, diarrhea, difficulty breathing, fatigue, fever, loss of smell and/or taste, or malaise).

This multi-institutional study was reviewed and approved by the institutional review boards of the University of Idaho (20-056, 20-060) and Tulane University (2020-602). All adult participants gave written informed consent for themselves and written assent for their infants.

### 2.2 RNA extraction

Total RNA was extracted from fecal samples using the Quick-DNA/RNA Viral MagBead kit (cat. R2140, Zymo Research). Briefly, ~100 mg of wet feces were mixed with 1000 µL of 1X DNA/RNA Shield (Zymo Research) and incubated for 10 min prior to extraction. Samples were then centrifuged for 2 min at 12,000 x g at 4°C, and RNA extracted from 300 µL of the supernatant following the manufacturer’s instructions, including a DNase I digest to remove double-stranded DNA. For negative and positive controls, fecal samples collected from healthy participants were left unspiked or spiked with heat-inactivated SARS-CoV-2 (isolate USA-WA1/2020, cat. NR-52285, BEI Resources), respectively.

### 2.3 Detection and quantification of SARS-CoV-2 RNA

RNA isolated from fecal samples (including negative and positive controls) was used as the input for Quick SARS-CoV-2 multiplex reverse-transcription quantitative polymerase chain reaction (RT-qPCR) assay (cat. R3013, Zymo Research). All RT-qPCR reactions were performed in duplicate. The Quick SARS-CoV-2 multiplex assay detects target sequences of the SARS-CoV-2 nucleocapsid (N) gene and the human RNase P gene. This multiplex assay was based on the US Centers for Disease Control and Prevention (CDC) protocol (42), and samples with Ct values <40 for the SARS-CoV-2 N gene were considered positive. RT-qPCR was performed with the Applied Biosystems 7500 Fast Real-Time PCR system with run mode set to “fast 7500” and cycling parameters set to run for 15 min at 55°C; 10 min at 95°C; followed by 5 sec at 95°C, 30 sec at 72°C, and 30 sec at 57°C for 45 cycles. A 5-point standard curve of SARS-CoV-2 RNA (cat. R3016, Zymo Research) was used to estimate viral load (range 80 to 50,000 genomic copies per reaction).

### 2.4 Statistical analyses

R (v4.2.2) (43) and GraphPad Prism 9 (v9.3.1; GraphPad Software) were used for data analyses. The R package ggplot2 (v3.4.0) (44) was used for data projection. The exact binomial test was used to calculate confidence intervals. Mann-Whitney tests for continuous data and Fisher’s exact tests for categorical data were used to test for differences in group characteristics. Fisher’s exact test was used to examine differences in the frequency of maternal/infant symptoms between groups, and associations between the presence of SARS-CoV-2 RNA in maternal/infant feces based on the presence of maternal/infant signs/symptoms. For the co-lactating maternal-infant dyad, a composite score of maternal SARS-CoV-2 positivity and symptomatology was created when assessing any relationships with infant outcomes; in all other instances the mothers were analyzed independently. Values are given as median ± interquartile range (IQR), unless otherwise indicated. Statistical significance was declared at *P*<0.05.

## 3 Results

### 3.1 General characteristics of participants and their infants

A total of 57 maternal-infant dyads provided maternal and/or infant fecal samples, including 33 dyads in the COVID-19 group and 24 dyads in the healthy group (**Table 1**). There were no differences between the groups with respect to maternal age, race/ethnicity, parity, time postpartum, infant sex, or breastfeeding exclusivity. Median age of adult participants was 32 yr (interquartile range: 30-35 yr), and they identified predominantly as white and non-Hispanic (88%). Median parity was two children; median infant age was 27 wk at baseline; half the infants were female; and 31% were exclusively breastfeeding. Of note, 11 infants of mothers in the COVID-19 group were also tested for COVID-19 prior to study enrollment, and 45% (*n*=5) of these infants received a positive COVID-19 diagnosis (**Table 1**).

**Table 1:** Selected baseline characteristics of study participants in the COVID-19 and healthy groups.

Characteristic	Total *n* (% or IQR)	COVID-19 *n* (% or IQR)	Healthy *n* (% or IQR)	*P*
Maternal participants, *n*	58^a^	34^a^	24	–
Age, median, yr	32 (30-35)	33 (30-36)	32 (28-34)	0.462
Race, ethnicity				0.159
White, Hispanic	4 (7)	4 (12)	0	
White, non-Hispanic	51 (88)	29 (85)	22 (92)	
Multiple^b^	3 (5)	1 (3)	2 (8)	
Maternal body mass index, median, kg/m^2^	27 (22-31)	26 (22-32)	27 (21-31)	0.493
18.5 to <25 (healthy)	23 (40)	14 (41)	9 (38)	0.889
25 to <30 (overweight)	15 (26)	8 (24)	7 (29)	
≥30 (obese)	20 (34)	12 (35)	8 (33)	
Parity, median, *n*	2 (1-2)	2 (1-3)	1 (1-2)	0.051
Infant participants, *n*	57	33	24	–
Infant age, median, wk	27 (15-42)	25 (15-33)	30 (15-44)	0.379
Infant sex, female, *n*	29 (51)	16 (48)	13 (54)	0.790
Exclusively breastfeeding^c^	18 (32)	14 (42)	4 (17)	0.048
Infant tested for COVID-19	11 (19)	11 (33)	–	–
Diagnosed with COVID-19^d^	5 (45)	5 (45)	–	–

IQR, interquartile range. Percentages may not sum to 100 due to rounding. P values calculated from Mann-Whitney tests for continuous data and Fisher’s exact tests for categorical data. ^a^ includes one co-lactating dyad. ^b^ includes American Indian/Alaskan and/or Latino/Hispanic and/or Mestizo and/or White, non-Hispanic. ^c^ includes provision of milk directly at the breast and/or expressed milk and no provision of non-breastmilk liquids (including water), formula, and/or complementary/solid foods. ^d^ percentage calculated by dividing the number of infants diagnosed with COVID-19 by the total number of infants tested for COVID-19.

### 3.2 Illness-related symptomatology of mothers and infants

**Figure 1:**
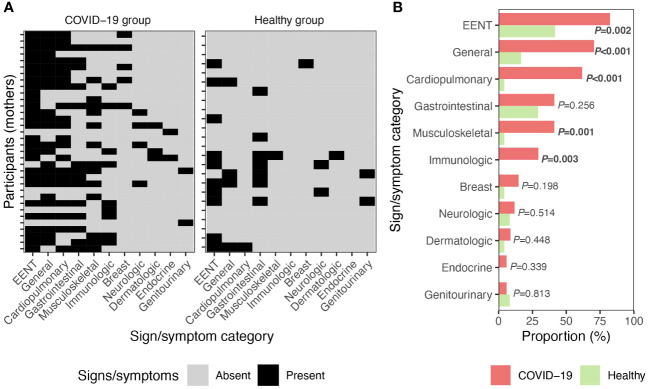
Signs/symptoms of illness in mothers. **(A)** Signs/symptoms reported by women in the COVID-19 (*n*=34) and healthy (*n*=24) groups. **(B)** Proportion of women diagnosed with COVID-19 (*n*=34) and self-reported healthy participants (*n*=24) with signs/symptoms. *P* values calculated with Fisher’s exact test. EENT, ears, eyes, nose, and throat.

The most commonly reported signs/symptoms among the women in the COVID-19 group included those associated with ears, eyes, nose, and throat (EENT; *n*=28; e.g., headache, loss of taste/smell, nasal congestion, sore throat); general symptoms (*n*=24; e.g., fatigue/malaise); and cardiopulmonary-related signs/symptoms (*n*=21; e.g., chest tightness, cough, difficulty breathing) (**Figure 1**). Compared to women in the healthy group, those in the COVID-19 group had higher frequency of signs/symptoms related to EENT (42 vs. 82%, *P*=0.002), general (17 vs. 71%, *P*<0.001), cardiopulmonary (4 vs. 62%, *P*<0.001), musculoskeletal (e.g., back pain, muscle/joint pain; 0 vs. 41%, *P*<0.001), and immunologic (e.g., chills, fever, sweating; 0 vs. 29%, *P*=0.003) variables (**Figure 1B**). There were no differences in the frequency of GI, breast, neurologic, dermatologic, endocrine, or genitourinary signs/symptoms between participants in the COVID-19 and healthy groups.

**Figure 2:**
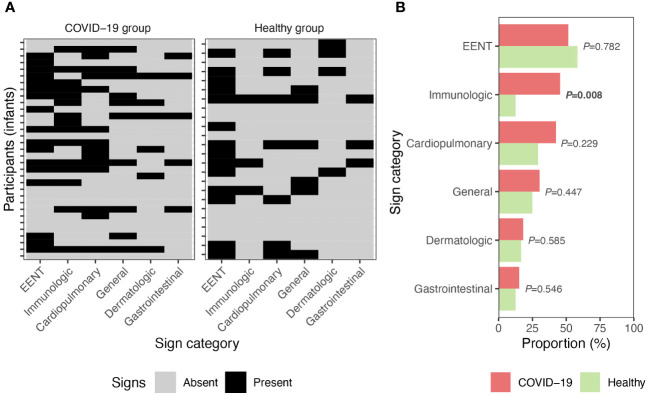
Signs of illness in infants. **(A)** Signs of illness reported by their parents for infants breastfed by women in the COVID-19 (*n*=33) and healthy (*n*=24) groups. **(B)** Proportion of signs present in infants within the COVID-19 and healthy groups. *P* values calculated with Fisher’s exact test. EENT, ears, eyes, nose, and throat.

In infants of mothers in the COVID-19 group, EENT, immunologic, and cardiopulmonary signs/symptoms were most common (**Figure 2**); however, rates of these signs/symptoms were similar to those reported for infants of mothers in the healthy group (**Figures 2B**). Compared to those in the healthy group, infants of mothers in the COVID-19 group had a higher frequency of immunologic symptoms (13 vs. 45%, *P*=0.008) (**Figure 2B**); this was solely attributed to fever.

### 3.3 Presence of SARS-CoV-2 in feces collected from women with and without COVID-19

**Figure 3:**
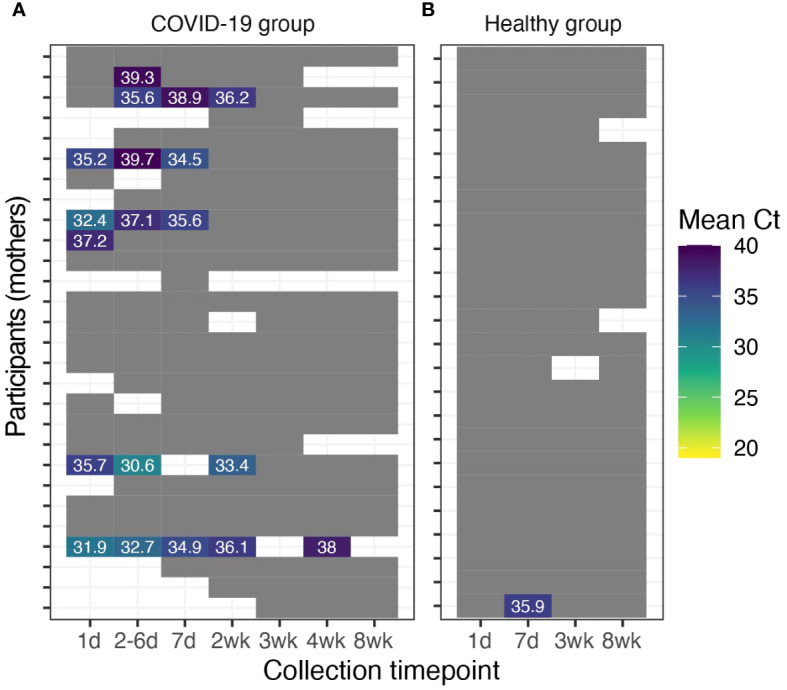
Detection of SARS-CoV-2 RNA in maternal fecal samples. Maternal fecal samples with detectable SARS-CoV-2 from participants **(A)** diagnosed with COVID-19 (*n*=28) and **(B)** healthy participants (*n*=24). Grey cells indicate fecal samples with no detectable SARS-CoV-2 RNA. White cells indicate uncollected/unavailable fecal samples.

A total of 28 (82.3%) women in the COVID-19 group provided fecal samples, which were collected 1-7 times over the 2-mo study period (**Figure 3A**; **Supplementary Table S1**, **Supplementary Figure S1**). SARS-CoV-2 RNA was detected in feces provided by seven (25%; 95% CI 10-45%) of these women. Five out of 19 (26%) fecal samples collected on the first day were positive for SARS-CoV-2 RNA, 6/21 (29%) at 2-6 d, 4/24 (17%) at 7 d, 3/25 (12%) at 2 wk, and 1/24 (4%) at 4 wk. SARS-CoV-2 RNA was detected in one sample collected from the 24 mothers in the healthy group who provided fecal samples. This sample was collected following enrollment on day 7 (**Figure 3B**).

### 3.4 Presence of SARS-CoV-2 in feces from breastfed infants of mothers with and without COVID-19

**Figure 4:**
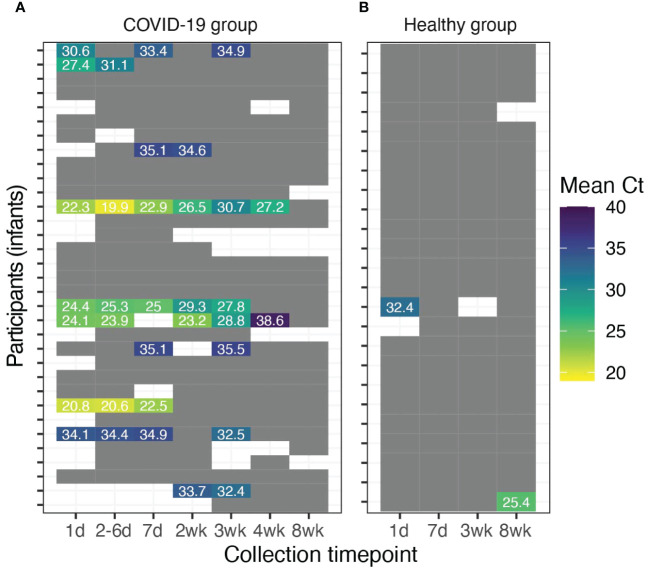
Detection of SARS-CoV-2 RNA in fecal samples of breastfed infants. Infant fecal samples with detectable SARS-CoV-2 RNA in **(A)** breastfed infants of mothers diagnosed with COVID-19 (*n*=33) and **(B)** breastfed infants of healthy mothers (*n*=24). Grey cells indicate fecal samples with no detectable SARS-CoV-2 RNA. White cells indicate uncollected/unavailable fecal samples.

Of the 33 infants of mothers in the COVID-19 group, 10 (30%, 95% CI 16-49%) had at least one sample that was positive for SARS-CoV-2 RNA (**Figure 4A**; **Supplementary Table S1**, **Supplementary Figure S1**). All 10 of these infants provided fecal samples out to 8 wk, at which time all were negative for SARS-CoV-2. In these infants fecal SARS-CoV-2 RNA shedding persisted for a duration ranging between 1 to 4 wks after enrollment. Specifically, 2 infants experienced viral RNA shedding for 1 wk, 1 infant for 2 wk, 5 infants for 3 wk, and 2 infants for 4 wk post-enrollment.

Among the 24 infants of women in the healthy group, two had single fecal samples positive for SARS-CoV-2 RNA (**Figure 4B**). Feces collected from one of these infants contained SARS-CoV-2 in the first week following enrollment. In the other case, although the infant’s first three samples (wk 1-3) were negative, the sample collected at the final timepoint (wk 8) was positive.

### 3.5 Intersection of maternal-infant fecal SARS-CoV-2 shedding

**Figure 5:**
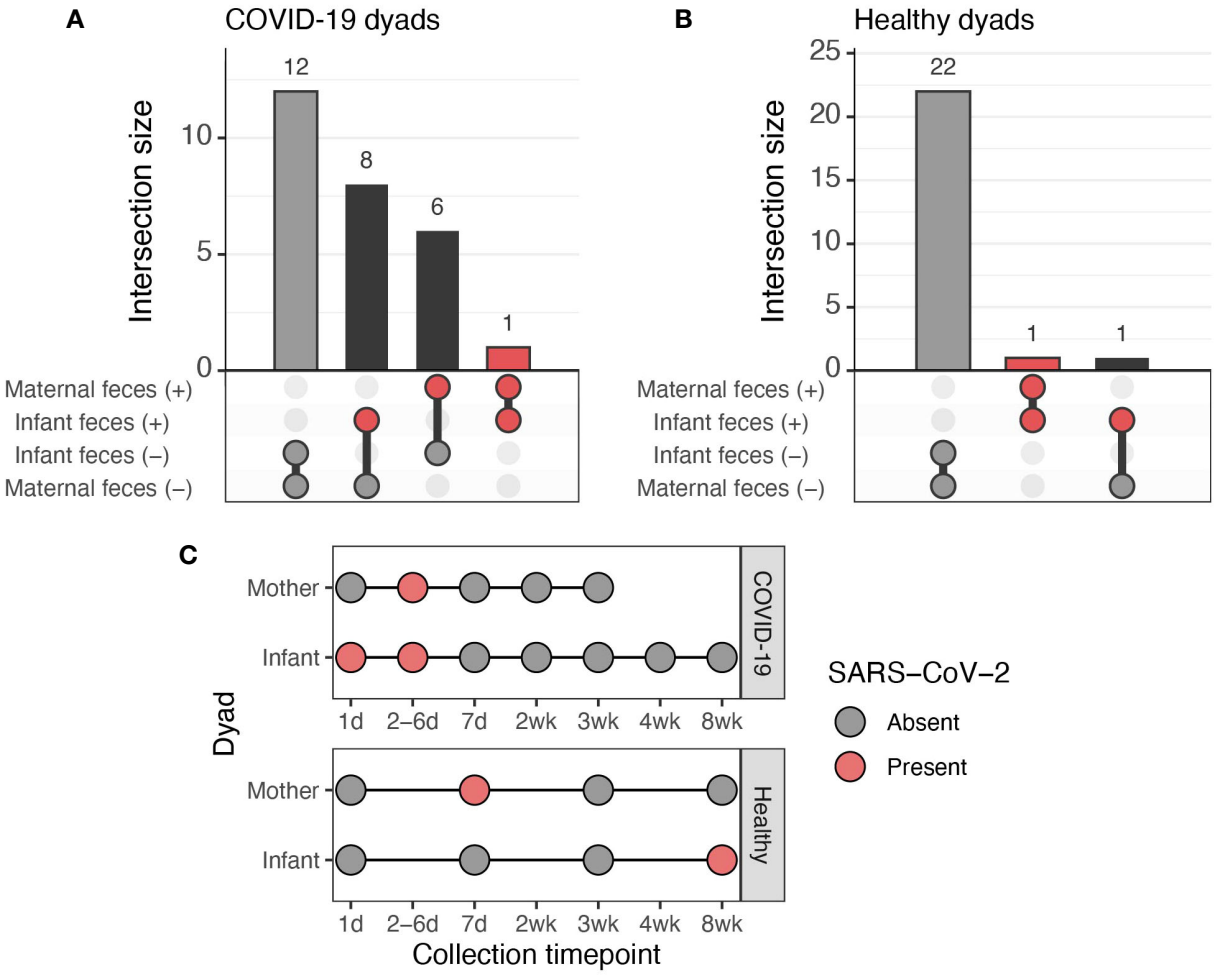
Co-occurrence of SARS-CoV-2 in feces of mothers and infants. **(A)** Upset plot of the 27 maternal-infant dyads in the COVID-19 group with paired fecal data. **(B)** Upset plot of the 24 maternal-infant dyads in the healthy group with paired fecal data. **(C)** Overview of fecal sampling and fecal SARS-CoV-2 results for the two dyads in which both the mother and infant had fecal samples positive for SARS-CoV-2.

Examination of SARS-CoV-2 positivity among the 27 maternal-infant dyads in the COVID-19 group that each provided at least one fecal sample revealed that 44% (*n*=12) of these dyads’ feces were negative for SARS-CoV-2 (**Figure 5A**). Only one dyads’ feces were simultaneously positive for SARS-CoV-2 (**Figure 5C**), and this only occurred once within the first week; all remaining samples thereafter testing negative. All other COVID-19 group dyads had discordant presence of SARS-CoV-2 in maternal and infant feces. In 22% (n=6) of dyads, the mother produced feces positive for SARS-CoV-2, whereas the infant did not. In the remaining 30% (*n*=8) of the dyads, the infants had feces containing SARS-CoV-2, whereas the mothers did not. Overall, there was no association between the presence of SARS-CoV-2 in maternal and infant feces in the COVID-19 group (*P*=0.36, Fisher’s exact test).

Most (92%, *n*=22) of the 24 maternal-infant dyads in the healthy group provided fecal samples that were consistently negative for SARS-CoV-2 (**Figure 5B**). One dyad was discordant for the presence of SARS-CoV-2, with one positive sample from the infant (first sample collected, “1 d”) and all negative samples from the mother. The mother and infant in the remaining dyad each had at one fecal sample positive for SARS-CoV-2, with the infant’s being collected in wk 8 and the mother’s during wk 1 (**Figure 5C**).

### 3.6 Associations between symptomatology and presence of SARS-CoV-2 in feces

We examined whether presence of maternal and/or infant signs/symptoms were related to the likelihood of SARS-CoV-2 positivity in fecal samples provided by mothers and their infants in the COVID-19. For mothers, there were no associations between the presence of EENT, general, cardiopulmonary, gastrointestinal musculoskeletal, or immunologic related signs/symptoms and presence of SARS-CoV-2 in their feces (**Table 2**). In contrast, the presence of immunologic symptoms in infants was associated with a 9-fold increased likelihood of SARS-CoV-2 in their feces (odds ratio [OR] 9.14 [95% CI 1.71-47.59], *P*=0.020; **Table 2**). Similarly, immunologic symptomology in the mother was associated with a 7-fold increased likelihood of SARS-CoV-2 in infant feces (OR 7.00, 95% CI 1.11-32.30; *P*=0.039; **Table 2**).

**Table 2:** Likelihood of SARS-CoV-2 detection in maternal/infant feces based on presence of maternal/infant signs/symptoms.

Signs/symptoms	Maternal feces based on maternal signs/symptoms		Infant feces based on infant signs		Infant feces based on maternal signs/symptoms	
	OR (95% CI)	*P*	OR (95% CI)	*P*	OR (95% CI)	*P*
Cardiopulmonary	0.75 (0.13-3.11)	>0.999	2.81 (0.64-10.61)	0.511	1.27 (0.22-5.80)	>0.999
EENT	0.80 (0.14-5.27)	>0.999	0.92 (0.20-4.25)	>0.999	2.29 (0.26-31.06)	0.636
Gastrointestinal	1.57 (0.30-7.21)	0.698	4.50 (0.75-27.84)	0.149	1.96 (0.34-8.37)	0.448
General	5.73 (0.63-70.68)	0.194	3.60 (0.67-14.66)	0.215	4.00 (0.50-50.93)	0.363
Immunologic	1.33 (0.32-7.36)	>0.999	9.14 (1.71-47.59)	0.020	7.00 (1.11-32.30)	0.039
Musculoskeletal	0.83 (0.21-4.08)	>0.999	–	–	1.00 (0.24-5.00)	>0.999

OR, odds ratio; CI, confidence interval; EENT, ears, eyes, nose, and throat. P values calculated with Fisher’s exact test, n=28 maternal feces based on maternal signs/symptoms; n=33 infant feces based on infant signs; n=27 infant feces based on maternal signs/symptoms.

## 4 Discussion

We assessed and characterized the frequency of SARS-CoV-2 fecal shedding and illness symptomatology across 57 breastfeeding maternal-infant dyads for 8 wk, including 33 dyads with sampling beginning within 7 d of maternal COVID-19 diagnosis (COVID-19 group) and 24 self-reported healthy dyads that served as controls (healthy group). Approximately 25% of women diagnosed with COVID-19 and their infants had detectable SARS-CoV-2 RNA in their feces (**Figures 3**, **4**). Among individuals with SARS-CoV-2 RNA detected in their feces, mean Ct values increased over time (i.e., decreased concentration of SARS-CoV-2), and SARS-CoV-2 RNA was undetectable in all fecal samples tested at 8 wk post-diagnosis except for one infant from the healthy group. Interestingly, in many cases, when SARS-CoV-2 RNA was detected, Ct values in infant feces were lower than the Ct values observed in the maternal fecal samples indicating higher possible higher viral loads. Although we extracted RNA from similar fecal amounts, differences in wet/dry weights and output mass of infant and maternal fecal samples may partially explain some of these differences in Ct values.

Not surprisingly, several groups of symptoms were reported by mothers diagnosed with COVID-19 (**Figure 1**). Compared to healthy controls, women with COVID-19 reported higher rates of general symptoms (e.g., fatigue/malaise), EENT, and cardiopulmonary, musculoskeletal, and immunologic signs/symptoms. This is consistent with the broad literature that has demonstrated individuals, including lactating women, with COVID-19 display an array of signs/symptoms stereotypical of respiratory virus infections (10, 14, 45–49).

The infants whose mothers were diagnosed with COVID-19 had similar rates of illness signs/symptoms compared to infants of the healthy mothers (**Figure 2**). The lone exception to this was that, compared to infants with healthy mothers, infants whose mothers were in the COVID-19 group had a higher proportion of immunologic symptoms, which were solely attributed to fever.

Among mothers, despite wide reporting of signs/symptoms, we found no associations between the presence of signs/symptoms of illness, including gastrointestinal, and detection of SARS-CoV-2 RNA in feces (**Table 2**). In infants, both infant and maternal immunologic-related symptoms (i.e., fever) were related to the detection of SARS-CoV-2 RNA in feces (**Table 2**).

Finally, we found no relationship between presence of SARS-CoV-2 RNA in maternal and infant feces (**Figure 5**). Analysis of paired maternal-infant dyad fecal samples indicated that SARS-CoV-2 RNA was absent in 44% of dyads. Only two maternal-infant dyads produced feces that contained SARS-CoV-2 RNA, although only one of these dyads (from the COVID-19 group) produced fecal samples that were concurrently positive (**Figure 5C**).

Interestingly, although mothers in the healthy group reported having no known contact with COVID-19 cases, the detection of SARS-CoV-2 RNA in the feces of one mother and her infant, as well as that of an unrelated infant, suggests that exposure had likely occurred unknowingly. This is in line with prior data from our group (41) and others on the presence of circulating anti-SARS-CoV-2-spike antibodies in blood samples collected from apparently healthy individuals during the pandemic. We do note that for the dyad with concordant fecal shedding, the mother tested negative for circulating anti-SARS-CoV-2 antibodies at 14-, 30-, and 60-d post-enrollment (circulating anti-SARS-CoV-2 antibody data was not available for the infant; results published previously (41)). Thus, an alternative explanation for this dyad’s results is that they are false positives and/or the fecal samples became inadvertently contaminated at some point in time, although we strictly adhered to containment and decontamination protocols during sample processing and analyses to prevent this scenario.

It is also worth noting that the SARS-CoV-2 RNA that was detected in the infants’ feces is unlikely to have come from their mothers’ milk as we have previously demonstrated that milk samples collected from this group did not contain SARS-CoV-2 RNA. These infants were likely infected via other routes, for example respiratory transmission. Importantly, none of these mothers or infants were hospitalized, and overall infants of mothers with COVID-19 had similar rates of signs of illness compared to infants of healthy mothers, except for an increased proportion of fever.

Findings from prior studies have found evidence of prolonged GI SARS-CoV-2 infections in individuals with symptomatic and asymptomatic COVID-19 (50–52). In many cases, GI symptoms (e.g., abdominal pain, nausea, and vomiting) in individuals with symptomatic COVID-19 are associated with fecal shedding of SARS-CoV-2 RNA (51). Our finding of SARS-CoV-2 RNA in maternal and infant feces during maternal COVID-19 are consistent with these findings. Despite our results, we did not detect a difference in the proportion of GI-related symptomatology in women with COVID-19 or their breasted infants compared to healthy controls.

There are several limitations to the current work. The sample sizes of the COVID-19 and healthy groups were relatively small, and COVID-19 diagnostic testing and results for infants during the study period were limited. Additionally, as most participants were recruited in the week following maternal COVID-19 *diagnosis* (and thus SARS-CoV-2 infection) we may have missed fecal shedding of SARS-CoV-2 in the immediate period following the onset of *infection*. Fecal shedding of viral RNA may have also occurred at levels below the limit of detection using RT-qPCR, although data suggest high concordance between RT-qPCR and the much more sensitive method of droplet digital PCR (51). Further, sample preservation and incomplete removal of inhibitors in fecal specimens may have impacted our ability to detect viral RNA (53, 54). Consequently, the results on the prevalence of fecal shedding in breastfeeding dyads should be interpreted to represent a lower bound due to the above. We also lacked serial naso/oropharyngeal sampling and therefore were unable to relate the duration of respiratory SARS-CoV-2 shedding with that in feces. Finally, as with all nucleic acid-based assays (e.g., RT-qPCR, PCR, nucleic acid sequencing), we were unable to determine whether any viral RNA detected came from infectious SARS-CoV-2 virions. Strengths of this study included the longitudinal nature of the experimental design, capture of maternal and infant symptomology, and rigorous and standardized sample collection methodology across study sites.

## 5 Conclusions

In this study, we found that fecal shedding of SARS-CoV-2 was relatively common in breastfeeding dyads (≥25% of lactating mothers and breastfeeding infants, respectively) in the 8 wk following maternal COVID-19 diagnosis, although the concordance of fecal shedding within the maternal-infant dyads was less common (~4%). Fecal SARS-CoV-2 shedding in mothers was not associated with any signs/symptoms. In contrast, infant fecal shedding was only associated with fever. Importantly and interestingly, our results do not support a relationship between presence of maternal and infant SARS-CoV-2 fecal shedding and maternal/infant SARS-CoV-2 infection. Together, these data indicate that feces are not a likely vehicle for the transmission of SARS-CoV-2 in breastfeeding dyads.

## Data availability statement

The original contributions presented in the study are included in the article/**Supplementary Material**. Further inquiries can be directed to the corresponding author.

## Ethics statement

The studies involving humans were approved by the institutional review boards of the University of Idaho (20-056, 20-060) and Tulane University (2020-602). The studies were conducted in accordance with the local legislation and institutional requirements. Written informed consent for participation in this study was provided by the participants’ legal guardians/next of kin. Written informed consent was obtained from the individual(s), and minor(s)’ legal guardian/next of kin, for the publication of any potentially identifiable images or data included in this article.

## Author contributions

RP: Conceptualization, Formal analysis, Funding acquisition, Investigation, Methodology, Project administration, Resources, Supervision, Visualization, Writing – original draft, Writing – review & editing. EK: Formal analysis, Investigation, Writing – review & editing. AM: Formal analysis, Investigation, Writing – review & editing. KP: Formal analysis, Investigation, Writing – review & editing. AW: Formal analysis, Investigation, Writing – review & editing. BC: Formal analysis, Investigation, Writing – review & editing. AN: Formal analysis, Investigation, Writing – review & editing. KL: Formal analysis, Investigation, Writing – review & editing. CP: Formal analysis, Investigation, Writing – review & editing. BF: Formal analysis, Investigation, Writing – review & editing. CS: Data curation, Formal analysis, Investigation, Writing – review & editing. MMc: Conceptualization, Formal analysis, Funding acquisition, Investigation, Methodology, Project administration, Resources, Supervision, Writing – review & editing. CB: Conceptualization, Formal analysis, Funding acquisition, Investigation, Methodology, Project administration, Resources, Supervision, Writing – review & editing. SL: Conceptualization, Formal analysis, Funding acquisition, Investigation, Methodology, Project administration, Resources, Supervision, Writing – review & editing. CM: Conceptualization, Formal analysis, Funding acquisition, Investigation, Methodology, Project administration, Resources, Supervision, Validation, Writing – review & editing. JW: Conceptualization, Formal analysis, Funding acquisition, Investigation, Methodology, Project administration, Resources, Supervision, Writing – review & editing. MAM: Conceptualization, Formal analysis, Funding acquisition, Investigation, Methodology, Project administration, Resources, Supervision, Writing – review & editing.
