# Supplementary material for: Prevalence and duration of SARS-CoV-2 fecal shedding in breastfeeding dyads following maternal COVID-19 diagnosis
Source: Front Immunol. 2024 Mar 21;15:1329092. doi: 10.3389/fimmu.2024.1329092 (PMC10996396; doi:10.3389/fimmu.2024.1329092)
Supplement: Supplementary file 1 [file DataSheet_1.docx]

Supplementary Material

Prevalence and duration of SARS-CoV-2 fecal shedding in breastfeeding dyads following maternal COVID-19 diagnosis

Ryan M. Pace^1,2^*, Elana A. King-Nakaoka^3,4^, Andrew G. Morse^3,4^, Kelsey J. Pascoe^5^, Anna Winquist^6^, Beatrice Caffé^7^, Alexandra D. Navarrete^8^, Kimberly A. Lackey^1^, Christina D.W. Pace^1^, Bethaney D. Fehrenkamp^1,3,4^, Caroline B. Smith^7^, Melanie A. Martin^9,10^, Celestina Barbosa-Leiker^5^, Sylvia H. Ley^11^, Mark A. McGuire^12^, Courtney L. Meehan^7^, Janet E. Williams^12^, Michelle K. McGuire^1^*

# Supplementary Data

## Supplementary Figures

**
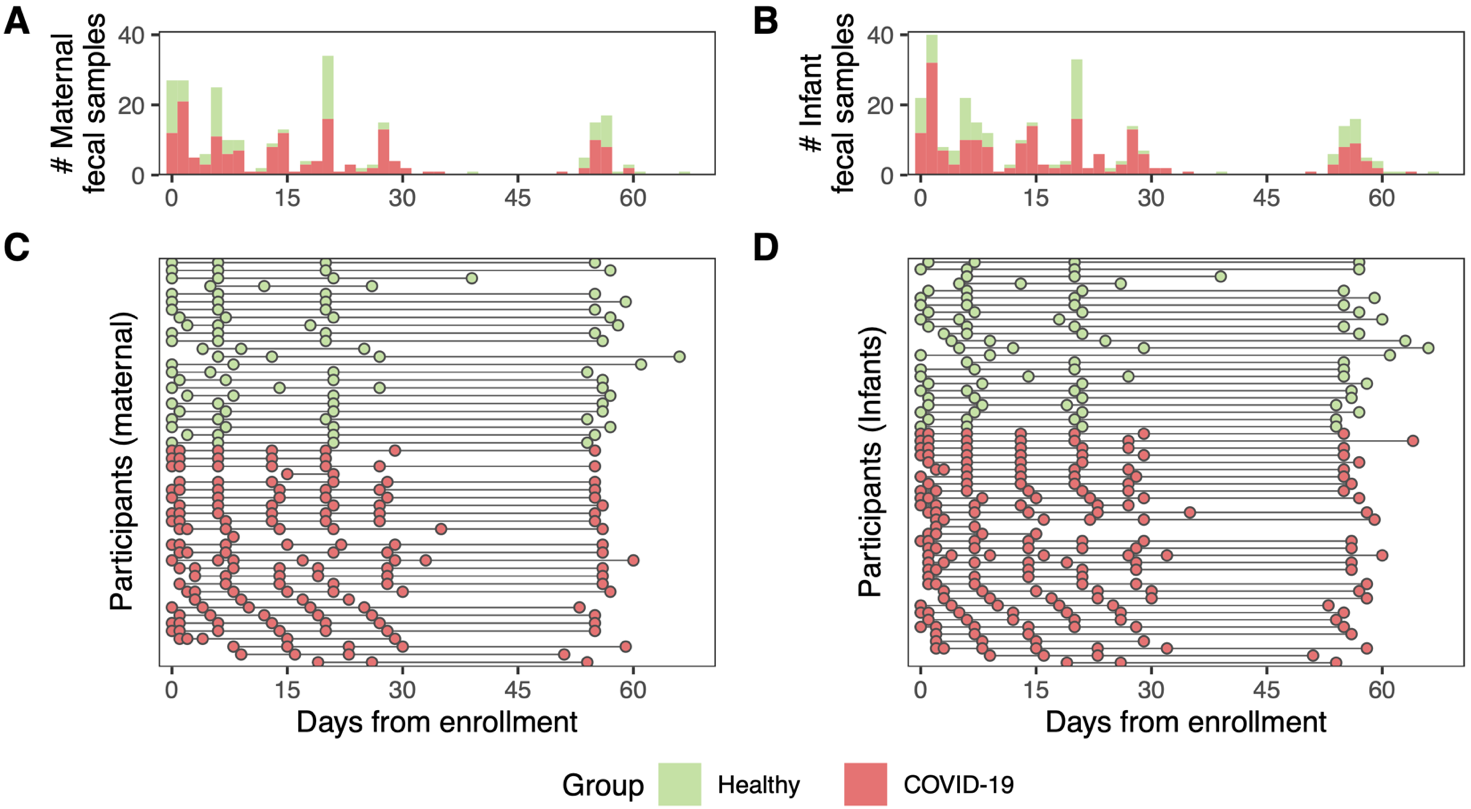
**

**Figure S1.** **Overview of maternal-infant fecal sample collections.** Distribution of fecal samples collected during the study from mothers (**A**) and infants (**B**), respectively, colored by group. Fecal samples collected during the study from individual mothers (**C**) and infants (**D**), respectively, colored by group.

## Supplementary Tables

**Table S1.** Overview of number of fecal samples collected during study from maternal-infant dyads in the COVID-19 and healthy groups.

|  | Total, *n* (%) | | COVID-19, *n* (%) | | Healthy, *n* (%) | |
| --- | --- | --- | --- | --- | --- | --- |
|  | Mothers | Infants | Mothers | Infants | Mothers | Infants |
| Enrolled, *n* | 58**^a^** | 57 | 34**^a^** | 33 | 24 | 24 |
| Provided ≥1 sample | 52 (90) | 57 (100) | 28 (82) | 33 (100) | 24 (100) | 24 (100) |
| Collection 1 - d 1 | 43 (74) | 45 (79) | 19 (56) | 22 (67) | 24 (100) | 23 (96) |
| Collection 2 - d 2-6 | 21 (36) | 30 (53) | 21 (62) | 30 (91) | - | - |
| Collection 3 - d 7 | 48 (83) | 53 (93) | 24 (71) | 29 (88) | 24 (100) | 24 (96) |
| Collection 4 - wk 2 | 25 (43) | 30 (53) | 25 (74) | 30 (91) | - | - |
| Collection 5 - wk 3 | 49 (84) | 52 (91) | 26 (76) | 29 (88) | 23 (96) | 23 (96) |
| Collection 6 - wk 4 | 24 (41) | 28 (49) | 24 (71) | 28 (88) | - | - |
| Collection 7 - wk 8 | 45 (78) | 51 (89) | 23 (68) | 28 (85) | 22 (92) | 23 (96) |
| Total number of samples | 255 | 289 | 162 | 196 | 93 | 93 |

Percentages may not sum to 100 due to rounding. ^a^ includes one co-lactating dyad.

**Table S2.** Grouping of signs/symptoms.

| Group | Signs/symptoms |
| --- | --- |
| Breast | Cancer, ectasia, mastitis |
| Cardiopulmonary | Cough, chest tightness, difficulty breathing, heart issue, pneumonia |
| Dermatologic | Acne, cellulitis, eczema, hives, rash |
| Ears, eyes, nose, and throat (EENT) | Allergies, conjunctivitis, ear infection, eye pressure/irritation, headache, loss of taste/smell, nasal congestion, postnasal drip, sneezing, sore throat |
| Endocrine | Hair loss, Hashimoto, hot flashes |
| Gastrointestinal | Constipation, diarrhea, flatulence, gastrointestinal discomfort/pain, loss of appetite, nausea, stomach pain, vomiting |
| General | Dehydration, disinterest in breastfeeding, fatigue, fussiness, insomnia, irritable, loss of appetite, low reactivity, malaise |
| Genitourinary | Bladder pain, urinary tract infection, uterine bleeding |
| Immunologic | Chills, fever, sweating |
| Musculoskeletal | Back pain, muscle/joint pain |
| Neurologic | Brain fog, dizziness, tingling sensation, vertigo |
